# Slow paced breathing and power posing: a pre-competitive ritual during an American football season

**DOI:** 10.3389/fspor.2026.1800382

**Published:** 2026-05-21

**Authors:** Erick Yael Fernández-Barradas, Luis Felipe Reynoso-Sánchez, Socorro Herrera-Meza, Jeanette M. López-Walle, Alma Gabriela Martínez-Moreno

**Affiliations:** 1 Institute for Research in Eating Behavior and Nutrition, University of Guadalajara, Ciudad Guzman, Mexico; 2 Faculty of Psychology, Veracruz University, Xalapa, Mexico; 3 Research Centre for Physical Culture Science and Health, Autonomous University of Occident, Culiacan, Mexico; 4 Faculty of Psychology, Autonomous University of Nuevo Leon, Monterrey, Mexico; 5 Institute of Psychological Research, Veracruz University, Xalapa, Mexico; 6 Faculty of Sport Organization, Autonomous University of Nuevo Leon, San Nicolas de los Garza, Mexico

**Keywords:** emotional regulation, intervention, IZOF model, pre-competitive anxiety, self-confidence

## Abstract

**Background:**

Psychological interventions before sports competition may be associated with changes in physical performance; however, the nature of sports practice usually limits the report of the benefits of psychology in this context. In this way, longitudinal studies offer a viable strategy for analyzing the impact of psychological preparation in competitive sports. Therefore, the objective of this study was to evaluate the potential influence of an intervention using slow-paced breathing and power posing on pre-competitive anxiety throughout a season in an amateur American Football team.

**Methods:**

A pre-experimental repeated-measures design with a single group was employed, consisting of a 9-week intervention. Thirty-four amateur athletes aged 16–18 years (*M* = 16.97, SD = .80) participated in the study. The Competitive State Anxiety Inventory-2 Revised (CSAI-2R) was administered before and after each game.

**Results:**

A reduction in one dimension of pre-competitive anxiety and a substantial increase in self-confidence were observed after the third week of the intervention.

**Conclusion:**

Findings are discussed in relation to the Individual Zones of Optimal Functioning (IZOF) model, highlighting the potential role of psychological techniques on emotional states preceding competition. The study concludes by proposing the development of longitudinal intervention protocols to monitor psychological processes across different sports disciplines.

## 1 Introduction

The ability to compete and manage complex situations during practice and competition has been a topic of interest in sports psychology (1–3). Currently, sports sciences have moved beyond analyzing physical, technical, or tactical dimensions and have integrated psychological skills as a dimension that significantly contributes to athlete performance and, therefore, may be linked to athletic success (4, 5). Among the psychological skills most studied in this field are the ability to stay focused during competition (6), motivational needs (7, 8), self-confidence (1) and even the influence of emotional state before a sport competition (9, 10). This has led to the study of how different psychological intervention techniques may affect these variables, improving the athlete's adaptive response to sports competition (2, 11–13).

The improvement of an athlete's psychological skills and their ability to adapt to any type of competition is one goal of the sport psychology training. However, standardizing sport psychology interventions is difficult because athletes and contexts are highly variable, and complex interventions must be adapted without losing their core active ingredients. According to literature (14, 15), most of the interventions are tested across many sports, levels, and outcome types, making any “average effect” hard to interpret in real-world terms. Nevertheless, there is evidence of the effectiveness that psychological interventions can have in improving the ability to manage emotions (16) communication processes (17), and even physiological variables (16, 18). In this way, some studies suggests that breathing techniques can have a significant impact on athletes' psychological state (19, 20), as well as the use of a pre-competition routine that can induce an optimal state for sports competition (21–23).

Slow-paced breathing (SPB) has been one of the most supported breathing techniques for modifying parasympathetic activity indicators (24), which has been associated with the ability to cope with stress (25, 26), manage emotions (27) and is even linked to the execution of complex cognitive tasks (28). SPB involves the use of the diaphragm to create breathing cycles of 10 s (4.5 s of inhalation followed by 5.5 s of exhalation) during sessions of 4, 8, 12, or even up to 16 min (26). This technique has been related to reductions of intrusive thoughts and may be linked to the induction of an optimal mental state prior to sports competition (29).

Prior research involving semi-professional adolescent swimmers (30) indicated that a 7-week slow-breathing intervention enhanced perceived performance, recovery status, and sense of control, although it did not significantly affect stress levels. Another example can be found in the study of Mosley et al. (31), who analyzed dual-career athletes who completed a 4-week smartphone-based slow-breathing program, reporting lower pre-performance anxiety, enhanced concentration, and improved relaxation before sleep. In addition, literature has reported that short bouts of slow breathing may enhance inhibitory control following physical exertion, which is particularly relevant for situations involving performance “under pressure” (32).

Another technique that appears to have interesting potential to influence the mental state prior to sports competition is Power Posing (PP), an exercise based on animal behavior and biology, involving expansive body postures that reflect dominance and power (33, 34). This exercise has been shown to be associated with physiological indicators such as increased testosterone levels (35) and even with changes in parasympathetic activation, on heart rate variability indicators (36). These physiological responses can be related to psychological variables such as self-confidence, reducing anxiety levels and achieving an optimal mental state prior to sports competition (35, 37). While there is no evidence to suggest that any specific posture directly improves sports performance, several postures have been reported to increase both confidence and physiological activation (33, 36, 37) which could contribute to an ideal state for managing competition-related stress.

Empirical research on PP has produced mixed results; initial experimental evidence suggested that brief adoption of high-power poses could lead to increased testosterone, decreased cortisol, and greater risk tolerance (34). Subsequent large-scale replication attempts failed to confirm these hormonal effects, but they consistently reported increases in subjective feelings of power and confidence (38). In this line, research suggest that power posing may influence self-efficacy, action readiness, and performance under stress through embodied cognition processes and not only direct endocrine changes (39, 40). In applied settings, adopting expansive postures has been associated with improved performance in stressful tasks and greater perceived control (41). From this perspective, power posing may function as a low-cost, easily implementable strategy to modulate pre-performance psychological states. When combined with techniques such as slow-paced breathing, which primarily target autonomic regulation, power posing may complement these effects by promoting approach-oriented states and self-confidence, thereby facilitating athletes' ability to reach their individual zone of optimal functioning rather than simply reducing anxiety or improve self-confidence.

The Individual Zone of Optimal Functioning (IZOF) is one theoretical model used to analyze the mental state before to sports competition (42), understanding how psychological states influence athletic performance. According to this model, athletes do not perform optimally at minimal levels of anxiety, it suggests that athletes have a range of physical and psychological activation in which they perform at their best. Both excessive and insufficient activation may impair performance, highlighting the importance of self-regulation strategies to achieve an optimal state prior to competition, and that it is possible to practice different psychological intervention techniques to reach this point before and during sports competition (43). Psychological intervention using IZOF model has been shown that approaches focused on emotional self-regulation may help athletes to understand their own emotional experiences and use them to approach their optimal functioning zone (44). Studies in this area, consider that pleasant and unpleasant emotions can be functional for performance depending on their intensity and individual interpretation (9, 43, 44). From this perspective, sport psychological interventions represent a valuable approach for teaching and training psychological skills that enhance athletic performance.

The use of these strategies to activate the IZOF is often referred as a “pre-competitive ritual” which consists of a combination of cognitive, emotional, and behavioral actions designed to help the athlete to achieve personal well-being and the optimal activation for sport performance (9, 21). In this line, slow-paced breathing and power posing may represent complementary mechanisms for regulating athletes' pre-competitive states. While slow-paced breathing facilitates down-regulation of physiological arousal and emotional distress (45), power posing may enhance activation and self-confidence (46). Therefore, the combined use of these strategies may allow athletes to adjust their psychological state toward their individual optimal zone, rather than simply reducing anxiety levels. This becomes particularly relevant in high-demand sport contexts, where athletes are required to constantly adjust their emotional and physiological responses to perform effectively.

Although there are multiple ways to categorize sports, one type of discipline that requires a high level of physiological activation combined with the ability to manage emotional states is collision sports (American football, ice hockey, rugby, etc.), whose main characteristic is the inherent contact with opponents at high intensities throughout the activity (47). This contact introduces potential health risks, which may be linked to increased stress levels in athletes and the potential experience of negative emotional states before, during, and after competition (13, 48). In this context, both slow-paced breathing and power posing may serve as viable psychological strategies for athletes to reach an optimal mental state before competition and perform at their best, regardless of the outcome.

Specifically, in American football, psychological skills such as the ability to regulate arousal and self-confidence have been linked to improved performance (49). Research has also shown that depressive symptoms can change before and after a competitive season (50), and that extra-sport factors, such as social support, play an important role in helping athletes cope with stressful events (51). While these findings are significant, most studies have employed non-experimental and/or cross-sectional designs, limiting the understanding of how psychological processes change over time or how athletes respond to a sustained psychological intervention throughout a competitive season.

As mentioned above, intervention in the field of sport psychology has been a complex issue due to the difficulties of specific sporting processes, in addition to the conditions of each sport discipline where injuries, extra-sport situations, or dropout from the discipline have a considerable impact on the results that can be obtained at the end of different psychological intervention protocols (2); Effective implementation requires adaptability (language, session length, sport-specific examples), but excessive adaptation can compromise fidelity and undermine the mechanisms of change; therefore, striking the right balance between adaptability and reliability is essential for an intervention that is both effective and grounded in scientific and methodological rigor (52). This equilibrium makes it difficult to present research with longitudinal designs (with more than two data collection points), in addition to the challenge of design intervention protocols during the competitive season; for this reason, there is a need to clearly identify which elements constitute essential, non-negotiable components and which can be flexibly adapted to ensure intervention effectiveness across different local contexts (52). In attention to this problem, the creation of an intervention protocol that combines both techniques (SPB and PP) is presented as an adequate alternative for manipulating the pre-competitive state during a competitive season, providing evidence to support the long-term changes from a psychological intervention.

Despite the growing body of literature on psychological skills in sport, most studies have relied on cross-sectional or short-term designs, limiting the understanding of how psychological interventions influence athletes over time. Furthermore, existing research has typically examined techniques such as slow-paced breathing and power posing in isolation, with little attention to their potential combined effects. Longitudinal intervention studies conducted during competitive seasons, particularly in collision sports, remain scarce due to the practical challenges of applied sport settings. This lack of ecologically valid research limits the ability to understand how integrated psychological strategies may influence athletes' pre-competitive states across a season.

Studying the pre-competitive state from a theoretical model such as the multidimensional model of pre-competitive anxiety becomes a key point to demonstrate the impact that psychological intervention techniques can have at the beginning, middle, and end of a regular season, generating an indirect indicator of cognitive, affective, and behavioral processes that affect athletes during the hours before to sports competitions. The main objective of this research was to evaluate the potential influence of an intervention with slow-paced breathing and power posing on pre-competitive anxiety throughout a sports season in an amateur American Football team. Based on previous empirical findings, SBP is expected to facilitate the regulation of arousal and emotional distress, whereas PP may enhance self-confidence and readiness for action. In combination, these strategies may contribute to the optimization of athletes' pre-competitive state. Accordingly, it was hypothesized that an intervention integrating SPB and PP would produce favorable longitudinal changes in athletes' pre-competitive psychological state across the competitive season. More specifically, cognitive and somatic anxiety were expected to decrease over time, while self-confidence was expected to increase from baseline to the subsequent assessment points.

## 2 Materials and methods

A quantitative, pre-experimental repeated measures study with a single group design was conducted, with treatment established over 9 weeks. This design allowed for the analysis of changes in self-confidence, cognitive anxiety, and somatic anxiety throughout a sports season with a total of 17 weekly assessments (pre- and post-game), including one training session during the week and one pre-competition session.

### 2.1 Participants

A non-probabilistic convenience sampling method was used, inviting all members of the sports team to participate in the study. The following inclusion criteria were used: (1) Being a member of the team; (2) Having at least 3 years of experience in the sport; (3) Attending 80% of the season's practices; (4) Participating in 100% of the season's games; (5) Participating in 100% of the intervention program sessions. The exclusion criteria were: (1) Pre-season sports injury; (2) Being a member of another sports club during the previous season; (3) Receiving psychological and/or psychiatric treatment; (4) Having a neuropsychological disorder. The elimination criteria were: (1) Suffering an injury during the competitive season; (2) Failing to complete any of the questionnaires. The final sample consisted of 34 American football players from Xalapa-Enríquez, Veracruz (Mexico), with an age range of 16–18 years (*M* = 16.97, SD = .80), all of whom were attending upper secondary education.

### 2.2 Data collection

#### 2.2.1 Pre-competitive anxiety

The Competitive State Anxiety Inventory—Revised (CSAI-2R) was used. This inventory contains a total of 17 Likert scale items that assess the intensity of physical sensations and the presence of cognitive content (0 = not at all; 4 = very much). The scale has seven items for the somatic anxiety dimension (e.g., “I feel very restless”); five for cognitive anxiety (e.g., “I am concerned about losing”); and five for self-confidence (e.g., “I feel confident about performing well”). This inventory is validated in the Mexican context (53). Internal consistency was assessed using baseline measurements with Cronbach's alpha and McDonald's Omega coefficients. For Cognitive Anxiety were *α* = .80, *ω* = .80; for Somatic Anxiety *α* = .85, *ω* = .86 and for Self-Confidence *α* = .85, *ω* = .85. Data were collected 16 times during the competitive season (including playoffs) and once during pre-season, generating a total of 17 measurements. The pre-competition anxiety questionnaire in its revised version was administered 48 h before and after each sports event, ensuring that it did not coincide with the weekly intervention session, except for session 1, where baseline data were collected.

#### 2.2.2 Slow paced-breathing and power posing intervention

The intervention protocol was designed by two sport psychologists with prior experience in the field. The protocol integrating elements from previously established slow-paced breathing (26) and power posing (36) protocols reported in the literature and adapting them to the specific demands of the competitive context. All sessions and intervention techniques were delivered in three phases by the sport psychologist responsible for the team (Table 1).

**Table 1:** Distribution of the sessions of intervention.

Timeframe	# Session	Format	Duration	Objective
Pre-season (week 1)	1	Weekly	60 min	Baseline setup and instruction of SPB and PP (40 min)
				Baseline assessment using CSAI-2R (10 min)
Regular season (week 2–7)	2/4/6/8/10/12	Weekly	60 min	SPB and PP (10 min)
				Teamwork/Cognitive Skills Exercise (30 min)
	3/5/7/9/11/13	Pre-competitive	10 min	SPB y PP (5 min)
				Game Plan
Playoff-semifinal (week 8)	14	Weekly	60 min	SPB and PP (10 min)
				Teamwork/Cognitive Skills Exercise (30 min)
				Regular season feedback (10 min)
	15	Pre-competitive	15 min	SPB and PP (7 min)
				Game Plan
Playoff-final (week 9)	16	Weekly	60 min	SPB and PP (10 min)
				Teamwork/Cognitive Skills Exercise (30 min)
				Game Plan (10 min)
	17	Pre-competitive	15 min	SPB and PP (7 min)
				Game Plan
Post-season (week 10)	18	Weekly	60 min	SPB and PP (10 min)
				Plenary session, end of intervention (30 min)

SPB, slow paced breathing; PP, power posing; CSAI-2R, competitive state anxiety inventory—revised.

##### 2.2.2.1 Phase 1: baseline measurement (week 1)

During this phase, all working sessions were scheduled, and the objectives and intervention techniques to be used were explained. SPB and PP were administered through facilitator-guided instructions combined with an auditory pacing stimulus indicating inhalation (4.5 s) and exhalation (5.5 s) with a diaphragmatic breathing cycle through nasal breathing (26). Simultaneously, participants adopted a standing high-power pose, with feet shoulder-width apart and hands resting on the hips, elbows naturally oriented outward, and the head slightly tilted backward (36). Both techniques were performed concurrently for 5 min with eyes closed.

##### 2.2.2.2 Phase 2: application of the intervention protocol (weeks 2–9)

16 sessions were distributed across 8 weeks (two sessions per week), with one session during the training week (72 h before the competition) and one pre-competitive session (60 min before the competition) Even-numbered sessions corresponded to weekly training sessions (60 min), whereas odd-numbered sessions were conducted prior to competition and consisted exclusively of a 5-minute SPB and PP practice (see Table 1).

Each weekly session lasted approximately 60 min and followed a consistent structure: SPB and PP practice at the beginning and end of the session, combined with team-building activities and cognitive skills exercises. Pre-competitive sessions were shorter (approximately 5 min) and involved only SPB and PP practice. Both techniques were applied following the same procedure described in Phase 1. Specifically, participants performed diaphragmatic breathing cycles (4.5 s inhalation, 5.5 s exhalation) guided by an auditory pacing stimulus and facilitator instruction, while simultaneously maintaining a standing high-power pose. Participants remained with eyes closed, using nasal breathing and focusing their attention on the breathing cycle.

Both techniques were applied simultaneously in a group setting for approximately 5 min in all sessions. All sessions were conducted by the same facilitator, and adherence to the intervention protocol was monitored through attendance records and session logs. Session delivery followed a standardized structure throughout the weeks.

##### 2.2.2.3 Phase 3: intervention closure and presentation of results (week 10)

At the end of the intervention protocol, participants received feedback on their performance throughout the season, and the results obtained were presented. Additionally, a plenary session was held to discuss performance during the competitive season.

This intervention protocol was planned for 1 week of pre-season, 6 weeks of the regular season (excluding playoffs), and one post-season week. The process was extended by two more weeks to include the semifinals and finals of the competitive season. All weekly and pre-competitive sessions were conducted on the American football field where the team trained and/or competed. For away games, the same intervention protocol was implemented under comparable conditions. Table 1 shows the distribution of the sessions conducted.

Weekly sessions' distributions and description are on Supplementary Table S1.

### 2.3 Procedure

This study was approved by a Research Ethics Committee (CONBIOÉTICA-30-CEI-006-20191210) under the reference number: 202308. The integrity and anonymity of participants' responses were ensured by following the ethical and procedural guidelines for research in sports medicine and exercise science (54). Once the project was approved, the team's authorities were first contacted to present the proposal. After receiving approval, all players were invited to participate in the study, and informed consent forms were distributed to guardians, along with assent forms for each athlete.

Following this, all intervention sessions were scheduled for the spring season (first semester) of 2024. The CSAI-2R inventory was administered 48 h before and after each game. At the conclusion of the intervention process, the results were presented to the sports institution, and feedback regarding performance during the sessions was provided to athletes and guardians upon request.

### 2.4 Data analysis

The data obtained were analyzed using the statistical software R Studio and JASP (Jeffrey's Amazing Statistical Software) for MacOS. Descriptive and inferential analyses were conducted, considering results with a *p*-value < .05 to be statistically significant. The Kolmogorov–Smirnov and Shapiro–Wilk tests were used to assess the normality of the data. Given that the data did not meet normality assumptions and considering the repeated-measures structure of the design, nonparametric methods were selected. According to this, the Friedman test for repeated measures was used to examine differences across the 17 measurement points throughout the competitive season. *Post-hoc* comparisons were conducted using Conover's test with Holm adjustment to control for Type I error. This approach allowed for a conservative evaluation of changes in psychological variables across time at the group level, consistent with the study design and objectives. Finally, the Friedman test for repeated measures was used along with the Conover *post hoc* test to identify significant differences between the data collection points throughout the competitive season.

## 3 Results

Table 2 shows the descriptive results for the variables cognitive anxiety, somatic anxiety, and self-confidence across all data collection points, from baseline to post-game eight evaluation.

**Table 2:** Descriptive statistics of the study variables pre- and post-competition during the competitive season.

Time	Moment	Self-confidence	Cognitive anxiety	Somatic anxiety
		*M* (SD)	*M* (SD)	*M* (SD)
Week 1	Baseline	15.44 (2.82)	15.29 (5.17)	15.14 (3.71)
Week 2	Pre	14.55 (2.17)	13.76 (4.00)	14.20 (4.49)
	Post	14.26 (2.65)	13.29 (4.45)	13.94 (4.86)
Week 3	Pre	13.35 (3.19)	13.44 (4.29)	13.97 (5.18)
	Post	15.73 (7.84)	12.23 (4.45)	14.02 (7.60)
Week 4	Pre	13.29 (3.26)	13.76 (4.34)	14.20 (4.78)
	Post	14.20 (2.61)	12.44 (4.30)	13.70 (4.62)
Week 5	Pre	14.02 (3.14)	11.88 (3.73)	13.67 (4.94)
	Post	14.11 (3.40)	12.26 (4.66)	14.17 (4.76)
Week 6	Pre	14.59 (3.86)	12.26 (5.27)	14.11 (5.48)
	Post	14.32 (3.40)	11.79 (4.48)	12.82 (5.31)
Week 7	Pre	14.23 (3.24)	12.38 (4.54)	13.26 (4.60)
	Post	14.52 (2.84)	12.52 (4.54)	13.55 (4.46)
Week 8	Pre	14.55 (2.85)	12.14 (4.37)	14.05 (5.29)
	Post	15.05 (2.87)	11.94 (4.66)	13.23 (4.79)
Week 9	Pre	14.61 (3.10)	12.38 (4.71)	14.64 (5.38)
	Post	12.76 (3.27)	11.76 (5.46)	14.70 (5.31)

SD, standard deviation; S, skewness; K, kurtosis.

Table 3 presents the results of the Friedman test for repeated measures. Given that the primary outcome of interest was pre-competitive state, *post-hoc* comparisons were conducted using Conover's test with Holm adjustment on pre-game measurements relative to baseline as these data may reflect athletes' emotional states prior to competition. In the case of Cognitive Anxiety, statistically significant differences were observed particularly in games 4, 6, 7 and 8. For Self-Confidence, non-statistically significant difference was detected on pre-game *post hoc* test (it only appears in G8 post). Finally, no statistically significant changes were detected for Somatic Anxiety.

**Table 3:** Friedman test of repeated measures with the variables cognitive anxiety, somatic anxiety, and self-confidence across 17 data points (baseline, 8 pre-game, 8 post-game).

Variable	*X* ^2^	df	*p*	Kendall's W	Conover's *post hoc* (vs. baseline)
Cognitive anxiety	52.27	16	< .001**	.09	Game 4 (pre)*
					Game 6 (pre)*
					Game 7 (pre)*
					Game 8 (pre)*
Somatic anxiety	19.73	16	0.23	.03	–
Self-confidence	38.06	16	.001**	.07	–

Only statistically significant *post-hoc* comparisons (Holm-adjusted) relative to baseline are presented for clarity; pre, pre-game measurement.

* *p* < .05.

** *p* < .01.

**Figure 1:**
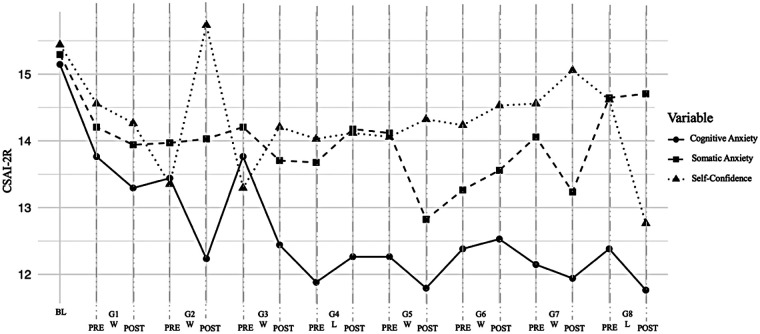
CSAI-2R scores throughout the competitive season considering baseline and pre-game and post-game data points. G#, game #; W, victory; L, loss.

The scores obtained on the pre-competitive anxiety scale (CSAI-2R), which visually corroborate the statistically significant differences reported in Table 3 (*p* < .05) for the Self-Confidence variable after game 2 and for the Cognitive Anxiety variable after game 3. In both cases, following the statistically significant difference, the scores stabilize, and no fluctuations are observed between the remaining data points (Figure 1).

Visual inspection suggests a progressive decrease in cognitive anxiety across the season, particularly from game 4 to game 8. This pattern is partially supported by the *post-hoc* test reported on Table 3, which indicates significant differences between baseline and pre-game measurements on games 4, 6, 7 and 8. In contrast, Somatic Anxiety shows a non-consistent directional pattern without statistically significant differences. Finally, self-confidence showed early-season fluctuations but appeared to stabilize after post-game 3 for the remainder of the season. A significant decrease was observed at post-game 8, which may be associated with the contextual outcome of the competition.

## 4 Discussion

### 4.1 Resource identification initiative

The main objective of this study was to evaluate the potential influences of slow-paced breathing and power posing intervention throughout a competitive season in an amateur American football team. Repeated measures analysis revealed changes associated with the intervention, reflected in a significant decrease in cognitive anxiety levels and stabilizes self-confidence levels across the season in the participants. These findings support the initial hypothesis and align with previous research highlighting the use of breathing techniques in reducing anxiety (18). Moreover, the observed increase in self-confidence may be related to the consistent use of power posing, as indicated in earlier studies (35).

These findings are consistent with the multidimensional model of pre-competitive anxiety, which considers self-confidence as a moderating variable capable of reducing anxiety levels (53). Therefore, the relative stability of self-confidence scores may be associated with the observed decrease in cognitive anxiety observed among the athletes in this study. This trend becomes apparent from mid-season, suggesting that the cognitive component of anxiety is related to confidence. It can be observed this perception apparent from game three, suggesting that the cognitive component of anxiety is inversely associated with confidence, where lower cognitive anxiety levels reflect higher self-confidence perception. This pattern aligns with what has been reported in cross-sectional designs, where regression analyses show that self-confidence becomes a key indicator that can influence other psychological variables essential for sports performance (1, 55).

When analyzing the pre- and post-game assessments, it is important to consider the timing of the measurements when interpreting these results. Assessments conducted 48 h before and after competition reflect aspects of the pre-competitive anxiety state, but do not fully represent immediate pre- or post-performance responses. In addition, these measurements may also capture broader emotional processes related to the anticipation of competition and post-event recovery.

It can be observed that somatic anxiety scores tend to be higher before the games than after. This may be primarily due to the close relationship between somatic components and physiological arousal reported in previous research (56). It may also be related to increased levels of perceived stress and physiological responses such as cortisol, or catecholamines (10), which the body releases to adapt to environmental demands, particularly in collision sports. As both conditions are necessary for individuals to face the challenges of athletic competition (3, 10, 57), the post-game decrease may be related to the exhaustion phase described in stress models, where the body begins its recovery period (58–60).

In the case of cognitive anxiety, several authors have reported that elevated scores in this dimension are to be expected prior to sports competitions (10, 19, 61). In this study, beyond confirming that pattern, a significant decrease in cognitive anxiety scores was observed over the course of the season. This may be associated to the implementation of slow-paced breathing (SPB) cycles. This technique has been related in previous research with increases in parasympathetic activity indicators, suggesting that SPB may improve regulation when facing stressful situations (18, 26). This also has been associated with reduced anxiety levels (20, 62) and may contribute to preventing the emergence of negative emotional states related to anxiety (16, 24). Additionally, completing a regular season with only one loss may have contributed to a cognitive restructuring process among the athletes, thereby decreasing the tendency to engage in negative thoughts about future sports performance. In this regard, it is important to consider that there are multiple factors that may influence the psychological variables assessed in this study, such as training experience, team cohesion, opponent level, and other individual characteristics. Therefore, the patterns observed in Figure 1 should not be interpreted as being exclusively attributable to the SPB and PP intervention, but as the result of the interaction between these contextual and performance-related factors.

Considering the self-confidence results, it can be observed that the scores for this variable tend to remain stable through the competitive season. This may be associated with physiological markers reported in the literature, such as changes on testosterone levels (35, 63) and changes in parasympathetic activity (36), which have been related with the use of power posing and may also be reinforced by the number of victories that led the team to the semifinals and finals of the season. Also, a significant decrease in self-confidence was observed at the end of the season, this may be associated with the outcome of the competition (loss a final game).

It is important to note that the associations between psychological processes and physiological mechanisms (e.g., parasympathetic activity, cortisol, or testosterone levels) are presented as theoretically informed interpretations based on literature, and not as direct evidence derived from the psychometric measures used in this study. These interpretations are grounded in the context of sport sciences, where performance-related outcomes are understood as multifactorial processes that involve the interaction of psychological, physiological, and contextual variables. From this perspective, linking psychological constructs with potential physiological mechanisms provides a conceptual framework for interpreting athletes' responses to competitive demands, even when such mechanisms were not directly assessed in the present study.

From a theoretical perspective, the IZOF model suggests that optimal athletic performance can be achieved within a continuum of both physical and mental activation, which is closely linked to anxiety states and the trait of self-confidence (38, 39). Although the model emphasizes that emotional experiences are highly individualized, the present study was based on group-level analyses and did not assess individual optimal functioning zones. Therefore, the IZOF model is used here as a conceptual framework to interpret the observed patterns. From this perspective, the shared context of practicing the same sport, training in coordinated sub-units, and competing as a team may contribute to the development of a collective optimal performance pattern. This is consistent with the observation that the average scores of the three variables remained relatively stable across different measurement points, while the outcomes were predominantly victories. These results align with patterns of consistent performance and highlight the potential relevance of pre-competition mental states in achieving athletic success.

Completing a sports season with a repeated measures design and a weekly intervention process combines two essential areas of sport psychology: intervention and research. While these areas are closely related, they often present certain challenges. On one hand, the complexity of intervention processes makes it difficult to generate scientific evidence that can be replicated and reported across different sports populations. On the other hand, cross-sectional, non-experimental studies with robust statistical analyses are more commonly observed, where inferences are made at a specific point in time. However, these inferences may not remain valid throughout a sports season, as it involves various factors that are crucial for understanding athletes' mental performance. Therefore, presenting a precedent that guides the integration of these two dimensions of sport psychology can serve as a starting point for developing scientific evidence that validates the effects of psychological interventions in this sports context.

### 4.2 Limitations and future directions

As a limitation, first, the sample size, with a small number of participants, may limit the magnitude and generalizability of the findings, regardless of the intervention design. Also, the participant selection criteria, which required athletes to have prior experience, high adherence to training, and full participation in the intervention, may have resulted in a relatively homogeneous and stable sample, potentially introducing selection bias and further limiting the generalizability of the findings to broader athletic populations. Additionally, in terms of the intervention design, there is no control group to compare the results of athletes who received the intervention with those who received no treatment. The study design does not allow for causal inferences regarding the observed changes in anxiety and self-confidence. In addition, the observed changes may also have been influenced by contextual factors inherent to a competitive season, such as performance outcomes, increasing familiarity with competition, team cohesion or coaching influences. Finally, although not a methodological limitation, losing the last game (conference final) may explain the considerable decrease in self-confidence. It would be interesting to have the opportunity to evaluate athletes who go on to win a championship.

One of the strengths of this study is the longitudinal design with repeated measures, which is one of the few studies in the field that had the opportunity to monitor psychological variables throughout an entire competitive season, considering both the intervention and the presence of weekly wins and losses. Additionally, the use of an easily accessible tool for assessment over short periods of time helped streamline the data collection process. It is important to note that, while the effects of some techniques have been previously reported in cross-sectional designs, this study combines two intervention techniques throughout a sports season, allowing the observation of psychological changes across time. Finally, these findings can serve as a foundation for future research to implement longitudinal designs with interventions that can further examine the role of mental preparation during different periods of the sports season.

It is suggested that future studies implement additional assessment tools that could be related to pre-competitive conditions (emotional states) and athletic performance (cognitive performance). In addition, the inclusion of biological markers such as heart rate variability, cortisol, or hormonal responses; would provide a more comprehensive understanding of the underlying mechanisms associated with psychological changes observed during the competitive season, as well as offer empirical support for the physiological interpretations discussed in this study. Furthermore, it would be valuable to include follow-up evaluations in subsequent seasons or compare a control group with an experimental group while assessing the same psychological variables.

## 5 Conclusion

In summary, the implementation of an intervention using cognitive-behavioral techniques such as SPB and PP was associated with changes in anxiety and self-confidence indicators throughout a sports season is evident. Additionally, the importance of the multidimensional model of pre-competitive anxiety and the Individual Zone of Optimal Functioning as theoretical models for understanding athletic performance across different longitudinal stages is highlighted. Finally, this study emphasizes the critical role of sport psychologists in providing support to athletes throughout a sports season.

## Data Availability

The raw data supporting the conclusions of this article will be made available by the authors, without undue reservation.
