# Supplementary material for: Slow paced breathing and power posing: a pre-competitive ritual during an American football season
Source: Front Sports Act Living. 2026 May 21;8:1800382. doi: 10.3389/fspor.2026.1800382 (PMC13233695; doi:10.3389/fspor.2026.1800382)
Supplement: Supplementary file 1 [file Datasheet1.pdf]

# *Supplementary Material*

## 1 Supplementary Tables

Table 1. Distribution of the sessions of intervention.

| Timeframe | *# Session* | *Format* | *Duration* | *Objective* |
|---|---|---|---|---|
| Pre-Season (Week 1) | 1 | Weekly | 60 min | Baseline setup and instruction of SPB and PP (40 min)<br>Baseline assessment using CSAI-2R (10 min) |
| Regular Season (Week 2-7) | 2/4/6/8/10/12 | Weekly | 60 min | SPB and PP (10 min)<br>Teamwork/Cognitive Skills Exercise (30 min) |
| | 3/5/7/9/11/13 | Pre-Competitive | 10 min | SPB y PP (5 min)<br>Game Plan |
| Playoff-Semifinal (Week 8) | 14 | Weekly | 60 min | SPB and PP (10 min)<br>Teamwork/Cognitive Skills Exercise (30 min)<br>Regular season feedback (10 min) |
| | 15 | Pre-Competitive | 15 min | SPB and PP (7 min)<br>Game Plan |
| Playoff-Final (Week 9) | 16 | Weekly | 60 min | SPB and PP (10 min)<br>Teamwork/Cognitive Skills Exercise (30 min)<br>Game Plan (10 min) |
| | 17 | Pre-Competitive | 15 min | SPB and PP (7 min)<br>Game Plan |
| Post-Season (Week 10) | 18 | Weekly | 60 min | SPB and PP (10 min)<br>Plenary session, end of intervention (30 min) |

Note: SPB = Slow Paced Breathing, PP = Power Posing, CSAI-2R = Competitive State Anxiety Inventory – Revised.

Table 2. Descriptive statistics of the study variables pre- and post-competition during the competitive season.

| Time | Variable | Mean | SD | S | K |
|---|---|---|---|---|---|
| Baseline (Week 1) | Self-confidence | 15.44 | 2.82 | -.09 | -.80 |
| | Cognitive Anxiety | 15.29 | 5.17 | -.47 | -.58 |
| | Somatic Anxiety | 15.14 | 3.71 | .25 | -.08 |

| | | *Pre* | | | | *Post* | | | |
|---|---|---|---|---|---|---|---|---|---|
| **Time** | **Variable** | **Mean** | **SD** | **S** | **K** | **Mean** | **SD** | **S** | **K** |
| Week 2 | Self-confidence | 14.55 | 2.17 | -.25 | -.52 | 14.26 | 2.65 | -.56 | .45 |
| | Cognitive Anxiety | 13.76 | 4.00 | -.06 | -1.17 | 13.29 | 4.45 | -.34 | -.99 |
| | Somatic Anxiety | 14.20 | 4.49 | .18 | -.76 | 13.94 | 4.86 | .46 | -.12 |
| Week 3 | Self-confidence | 13.35 | 3.19 | -.43 | -.30 | 15.73 | 7.84 | 4.97 | 27.32 |
| | Cognitive Anxiety | 13.44 | 4.29 | -.38 | -.81 | 12.23 | 4.45 | .20 | -.86 |
| | Somatic Anxiety | 13.97 | 5.18 | .62 | .54 | 14.02 | 7.60 | 2.22 | 5.80 |
| Week 4 | Self-confidence | 13.29 | 3.26 | -.24 | -.49 | 14.20 | 2.61 | -.06 | -.91 |
| | Cognitive Anxiety | 13.76 | 4.34 | -.35 | -.88 | 12.44 | 4.30 | .13 | -.84 |
| | Somatic Anxiety | 14.20 | 4.78 | .55 | .53 | 13.70 | 4.62 | .56 | .09 |
| Week 5 | Self-confidence | 14.02 | 3.14 | -.46 | -.37 | 14.11 | 3.40 | -.90 | .07 |
| | Cognitive Anxiety | 11.88 | 3.73 | .19 | -.08 | 12.26 | 4.66 | -.00 | -.94 |
| | Somatic Anxiety | 13.67 | 4.94 | .74 | .20 | 14.17 | 4.76 | .05 | -1.02 |
| Week 6 | Self-confidence | 14.59 | 3.86 | -1.24 | 1.75 | 14.32 | 3.40 | -.68 | .05 |
| | Cognitive Anxiety | 12.26 | 5.27 | -.200 | -.60 | 11.79 | 4.48 | .01 | -.97 |
| | Somatic Anxiety | 14.11 | 5.48 | -.00 | .27 | 12.82 | 5.31 | .82 | .12 |
| Week 7 | Self-confidence | 14.23 | 3.24 | -.37 | -.56 | 14.52 | 2.84 | -.18 | -.89 |
| | Cognitive Anxiety | 12.38 | 4.54 | .12 | -.57 | 12.52 | 4.54 | -.01 | -.95 |
| | Somatic Anxiety | 13.26 | 4.60 | .36 | -.52 | 13.55 | 4.46 | .17 | -.26 |
| Week 8 | Self-confidence | 14.55 | 2.85 | -.14 | -1.36 | 15.05 | 2.87 | -.40 | -1.17 |
| | Cognitive Anxiety | 12.14 | 4.37 | .08 | -.69 | 11.94 | 4.66 | .16 | -.97 |
| | Somatic Anxiety | 14.05 | 5.29 | .45 | -.16 | 13.23 | 4.79 | .44 | -.63 |
| Week 9 | Self-confidence | 14.61 | 3.10 | -.47 | -1.04 | 12.76 | 3.27 | -.39 | -.07 |
| | Cognitive Anxiety | 12.38 | 4.71 | -.16 | -1.34 | 11.76 | 5.46 | .15 | -1.43 |
| | Somatic Anxiety | 14.64 | 5.38 | .17 | -.92 | 14.70 | 5.31 | -.00 | -.98 |

Note: SD = Standard Deviation, S = Skewness, K = Kurtosis

Table 3. Friedman test of repeated measures with the variables Cognitive Anxiety, Somatic Anxiety, and Self-confidence across 17 data points (baseline, 8 pre-game, 8 post-game).

| Variable | Kendall's W | Conover |
|---|---|---|
| Self-confidence | .09** | Game 3 Post** |
| Cognitive Anxiety | .03 | ----- |
| Somatic Anxiety | .07** | Game 2 Post* |

Note: * = $p < .05$, ** = $p < .01$.

## 2 Supplementary Figures

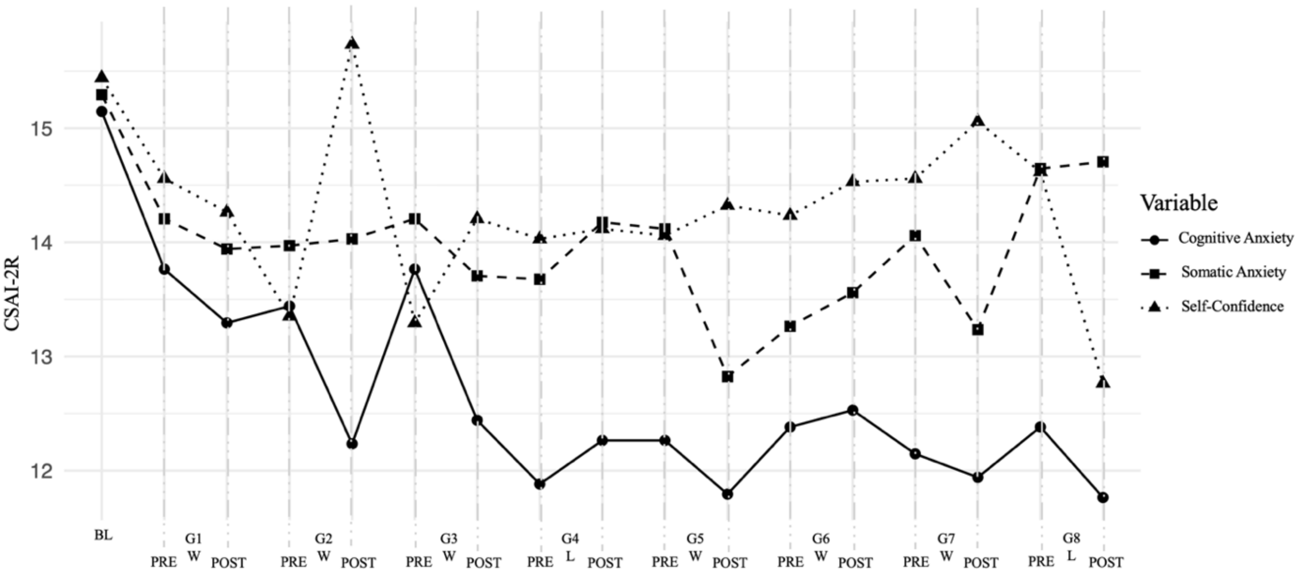


Note: G# = Game #, W = Victory, L = Loss.

**Supplementary Figure 1.** CSAI-2R scores throughout the competitive season considering baseline and pre-game and post-game data points.
