# Supplementary material for: Slow paced breathing and power posing: a pre-competitive ritual during an American football season
Source: Front Sports Act Living. 2026 May 21;8:1800382. doi: 10.3389/fspor.2026.1800382 (PMC13233695; doi:10.3389/fspor.2026.1800382)
Supplement: Supplementary file 2 [file Datasheet2.pdf]

Table S1. Distribution of weekly sessions of intervention.

Timeframe	***# Session***	***Additional Activities***
Pre-season (Baseline)	1	1. Team introduction<br>2. SPB instruction and practice<br>3. Power posing instruction and practice<br>4. CSAI-2R administration
Regular Season	2	1. SPB and PP practice (5 min).<br>2. Goal setting for competitive season (10 min).<br>3. Team-building activity 1 (30-40 min):<br>Participants engaged in a collaborative task in which they used recycled materials to construct a pathway to traverse 50 yards of the American football field in the shortest possible time. If any participant stepped off the pathway, the entire group was required to restart the task from the beginning. At the end of the activity, a brief facilitator-led feedback session was conducted, emphasizing the importance of teamwork, communication, and collective responsibility in sport performance.<br>4. SPB and PP practice (5min).
	4	1. SPB and PP practice (5 min).<br>2. Post-game feedback and goal setting review (10 min).<br>3. Team-building activity 2 (30-40 min):<br>Participants worked collaboratively in small groups to traverse 50 yards of the American football field while collectively carrying a football, ensuring that all group members maintained physical contact with the ball at all times. The activity began with groups of three participants, progressively increasing in size until the entire team completed the task together. At the end of the activity, a facilitator-led feedback session was conducted, emphasizing the importance of collaboration, shared responsibility, and collective goal pursuit in sport performance.<br>4. SPB and PP practice (5min)
	6	1. SPB and PP practice (5 min).<br>2. Post-game feedback and goal setting review (10 min).<br>3. Cognitive Skills Exercise 1 (30-40 min):<br>Participants performed individual passing routes at short (10 yards), medium (20 yards), and long distances (30+ yards). Each distance was associated with an auditory cue: one whistle for short routes, two consecutive whistles for medium routes, and three consecutive whistles for long routes. Participants were aligned in an offensive formation and initiated their routes based on the auditory signals. The quarterback (QB) selected the target receiver and executed the pass. At the end of the activity, a facilitator-led feedback session was conducted, focusing on attentional processes and decision-making during sport performance.<br>4. SPB and PP practice (5min).
	8	1. SPB and PP practice (5 min).<br>2. Post-game feedback and goal setting review (10 min).<br>3. Cognitive Skills Exercise 2 (30-40 min):<br>This activity built upon the previous session, maintaining a similar structure. Participants worked in small groups (rotations of three players) to execute short, medium, and long routes in response to auditory cues. Additional obstacles were incorporated on the field, increasing task complexity. The quarterback (QB) was required to select the safest passing option among the available receivers and execute the pass accordingly. At the end of the activity, a facilitator-led feedback session was conducted, focusing on attentional demands, decision-making, and adaptive responses under dynamic conditions.<br>4. SPB and PP practice (5min).

	10	1. SPB and PP practice (5 min).<br>2. Post-game feedback and goal setting review (10 min).<br>3. Cognitive skills exercise and team building activity 1 (30-40 min).<br>Participants engaged in a competitive task between offensive and defensive units, organized in groups of five players (5 vs. 5). Each group was required to traverse 50 yards of the American football field while carrying a ball using a tensioned fabric sheet. Every 10 yards, an additional ball was passed through the air and had to be caught using the same fabric sheet without dropping the initial ball. The round concluded when one of the teams successfully completed the distance while maintaining five balls on the fabric sheet. At the end of the session, a facilitator-led feedback discussion was conducted, emphasizing the importance of sub-unit coordination, sustained attention during gameplay, organization, and teamwork in achieving shared performance goals.<br>4. SPB and PP practice (5min).
	12	1. SPB and PP practice (5 min).<br>2. Post-game feedback and goal setting review (10 min).<br>3. Cognitive skills exercise and team building activity 2 (30-40 min).<br>The same task (session #10) structure was maintained with the addition of auditory stimuli and performance-based constraints. Instead of receiving a pass every 10 yards, participants were required to stop their movement upon hearing two consecutive whistle signals, at which point an additional ball was provided and incorporated into the task. The group then resumed the movement while maintaining all balls on the tensioned fabric sheet. If any sub-unit of five participants dropped a ball at any point, the group was required to restart the entire distance from the beginning. A facilitator-led feedback discussion followed the activity, focusing on attentional control, adaptability to external stimuli, and collective responsibility under increased task demands.<br>4. SPB and PP practice (5min).
Playoff Semifinal	14	1. SPB and PP practice (5 min).<br>2. Post-game feedback and goal setting review (10 min).<br>3. Cognitive skills exercise and team building activity 3 (30-40 min).<br>Participants were positioned with approximately 2 meters of distance between each player, and each participant was provided with a 50 ml plastic cup. The task consisted of passing a total of 34 table tennis balls through the air from one cup to another across the entire group sequence. If any ball was dropped during the process, it was returned to the starting point, and the sequence had to begin again. At the end of the session, a facilitator-led feedback discussion was conducted, emphasizing how sustained teamwork can lead to the achievement of collective goals, represented by the successful transfer of all 34 balls (one per participant). The discussion also highlighted the importance of self-regulation, attentional focus, and coordinated effort in tasks involving fine motor skills, as well as in more complex activities such as American football performance.<br>4. SPB and PP practice (5min).
Playoff Final	16	1. SPB and PP practice (5 min).<br>2. Post-game feedback and goal setting review (10 min).<br>3. Team building activity 3 (30-40 min).<br>All participants were invited to sign a team jersey as a symbolic gesture of shared experience throughout the season. Following this, team captains formally presented the jersey to the head coach as an expression of appreciation for the season. A facilitator-led reflection was conducted, framing the jersey as a symbol of commitment, effort, and teamwork. The session concluded with a collective group applause, reinforcing team cohesion.<br>4. SPB and PP practice (5min).
